# Football-specific validity of TRACAB’s optical video tracking systems

**DOI:** 10.1371/journal.pone.0230179

**Published:** 2020-03-10

**Authors:** Daniel Linke, Daniel Link, Martin Lames

**Affiliations:** Chair of Performance Analysis and Sports Informatics, Technical University Munich, Munich, Germany; Univ Rennes, FRANCE

## Abstract

The present study aimed to validate and compare the football-specific measurement accuracy of two optical tracking systems engineered by TRACAB. The “Gen4” system consists of two multi-camera units (a stereo pair) in two locations either side of the halfway line, whereas the distributed “Gen5” system combines two stereo pairs on each side of the field as well as two monocular systems behind the goal areas. Data were collected from 20 male football players in two different exercises (a football sport-specific running course and small-sided games) in a professional football stadium. For evaluating the accuracy of the systems, measures were compared against simultaneously recorded measures of a reference system (VICON motion capture system). Statistical analysis uses RMSE for kinematic variables (position, speed and acceleration) and the difference in percentages for performance indicators (e.g. distance covered, peak speed) per run compared to the reference system. Frames in which players were obviously not tracked were excluded. Gen5 had marginally better accuracy (0.08 m RMSE) for position measurements than Gen4 (0.09 m RMSE) compared to the reference. Accuracy difference in instantaneous speed (Gen4: 0.09 m⋅s^-1^ RMSE; Gen5: 0.08 m⋅s^-1^ RMSE) and acceleration (Gen4: 0.26 m⋅s^-2^ RMSE; Gen5: 0.21 m⋅s^-2^ RMSE) measurements were significant, but also trivial in terms of the effect size. For total distance travelled, both Gen4 (0.42 ± 0.60%) and Gen5 (0.27 ± 0.35%) showed only trivial deviations compared to the reference. Gen4 showed moderate differences in the low-speed distance travelled category (-19.41 ± 13.24%) and small differences in the high-speed distance travelled category (8.94 ± 9.49%). Differences in peak speed, acceleration and deceleration were trivial (<0.5%) for both Gen4 and Gen5. These findings suggest that Gen5’s distributed camera architecture has minor benefits over Gen4’s single-view camera architecture in terms of accuracy. We assume that the main benefit of the Gen5 towards Gen4 lies in increased robustness of the tracking when it comes to optical overlapping of players. Since differences towards the reference system were very low, both TRACAB’s tracking systems can be considered as valid technologies for football-specific performance analyses in the settings tested as long as players are tracked correctly.

## Introduction

Spatiotemporal data measured by Electronic Performance Tracking Systems (EPTS) has received increasing attention in research and practice. It can be used to derive performance indicators which are nowadays considered as an essential component in load monitoring [1] and tactical analysis [2]. EPTS can be differentiated based on the underlying measurement principle into three groups: Global Navigation Satellite Systems (GNSS; e.g., Global Positioning System GPS), radio-based local positioning systems (LPS) or video tracking systems [1]. In order to collect positional data during official match-play, the worlds’ top leagues such as Bundesliga, La Liga, Premier League or Ligue 1, rely on optical tracking systems [1]. One reason is that these systems provide the only non-intrusive solution to track both players and ball simultaneously without physical intervention by technical equipment. A second advantage is that there is no data loss due to sensor failure since data can always be restored from video footage. Thirdly, GNSS based systems also have the problem that they are less accurate in large stadia since the satellites required for positioning are not always visible.

Consequently, companies such as STATS (Chicago, US), Second Spectrum (Los Angeles, US), ChyronHego (New York, US), and Deltatre (Torino, Italy) have all provided video tracking systems to the market which allow positional data to be collected and used for live and post-match analysis, talent scouting, and media enhancement [3]. A comprehensive survey of the state-of-the-art video-based player tracking systems can be found in Manafifard, Ebadi [4], and a survey on football video analysis was provided by Oskouie, Alipour [5]. Worth mentioning in this context is that a variety of different video tracking systems exist, which can be broadly classified according to the number, arrangement, and specification of utilised cameras (single vs multiple, stationary vs dynamic, stereo vs monocular) [4].

The ever-increasing volume of available tracking data has also benefited the sports scientific community, leading to a marked increase in the number of publications investigating the use of tracking systems on measuring player’s physical performance during both match and training [6]. However, to allow meaningful interpretation of the results, scientists and practitioners inevitably face the challenge of understanding the validity and reliability of the used tracking data, especially when planning interventions [7]. Therefore, the data obtained by any tracking system should ideally be compared with the data of a reference system with known error estimates, also referred to as ‘gold standard’ validation concept [8].

This study is the first to evaluate the accuracy, validity and reliability of ChyronHego’s TRACAB^®^ system, which is one of the market leaders in the field of optical tracking systems. There are two versions available: the 4^th^ generation TRACAB^®^ system (Gen4) consists of two multi-camera units (stereo pair) in two locations either side of the halfway line, whereas the 5^th^ generation TRACAB^®^ system (Gen5) is a distributed camera system (combining two stereo pairs on each side of the field as well as two monocular systems behind the goal areas). Since 2013, Gen4 has tracked over 10.000 matches in professional soccer and is used in over 200 stadia, including the German Bundesliga, English Premier League, Spanish LaLiga, Dutch Eredivisie, Danish Superliga, as well as European matches in the UEFA Champions’ League and International matches in UEFA and FIFA tournaments. Gen5 has been available since 2019, is installed in 36 stadia of German Bundesliga and has tracked over 500 matches so far. There have been many scientific studies based on TRACAB’s data, dealing with individual ball possession [9], fatigue development [10], match half variation [11] and seasonal variation [12]. Others focussed on the pressure on players [13], situation assessment [14], attacking [15], space control [16], pressure [13], and free-kick performance [17]. However, all these studies share the limitation, that there is no validation available proving the quality of TRACAB^®^ data.

Accordingly, analysis of validity and reliability of EPTS has been subject to extensive research in recent years. While a large and ever-increasing number of GNSS and LPS validation studies can be found [7, 8, 18–22], only a few studies are available that validated the measurement accuracy of football-specific video tracking systems. To date, two studies fulfil this criterion. One study compared the average velocities delivered by Prozone^®^ (Leeds, UK; acquired by STATS LLC in 2015) with the average velocity derived from timing gates and reported typical error estimates between 0.1 and 5.5% [23, 24]. Using a similar methodology, Redwood-Brown, Cranton [25] analysed Venatrack^®^ (Venatrack, UK) and found average error estimates between 0.1 and 2.8%.

A limitation of these studies is that timing gates are only of limited suitability as a speed reference [26] as this approach merely determines average velocities based on limited sampling points [27]. Instead, a favourable validation approach is to compare the instantaneous kinematics (position, velocity and acceleration) with that of a two or three-dimensional reference system [8]. To the best of our knowledge, however, the only football-specific video tracking system that has been validated using such a criterion measure approach is the SportVU system (Stats Perform, US) [28]. Using a VICON motion capture system as criterion reference, we found that SportVU underestimated the total distance by 0.57%, high-speed running (20 to 25 km⋅h^-1^) by 11.35%, and very high-speed running (>25 km⋅h^-1^) by 14.31%. These recent results have yet again confirmed that, despite many years of advancements in computer vision and tracking algorithms, optical player detection and tracking are still quite challenging due to many difficulties such as similar appearance of players, occlusions, weather-related background changes, varying number of players with unpredictable movements, calibration inaccuracy, noise, clutter, motion blur, and a lack of pixel resolution especially on small distant players [4].

Against this background, the primary aim of the study was to determine TRACAB’s accuracy of spatiotemporal tracking variables in a football-specific environment. Since tracking systems differ significantly in terms of the applied camera setup, sampling rates and data processing steps, validation results of one system cannot be extended to others [27]. Instead, individual validation of each system is required [21, 25]. A secondary aim of the study was to assess whether there is a difference between the Gen4 and the Gen5 system. As a multi-camera system, Gen5 has potential benefits (e.g. less tracking loss, more pixels available), but it is unclear if accuracy also benefits from multiple views. To address these questions, measures of each system were compared to simultaneously recorded measures of a criterion reference (VICON). The results provide a better understanding of the validity and reliability of the growing body of TRACAB data.

## Materials and methods

In line with the aims of our study, we compared data of TRACAB against simultaneously recorded measures of a criterion reference technology with superior accuracy and known error estimates. This was done for test runs along predefined tracks and small-sided games (SSG).

### Venue and participants

**Fig 1:**
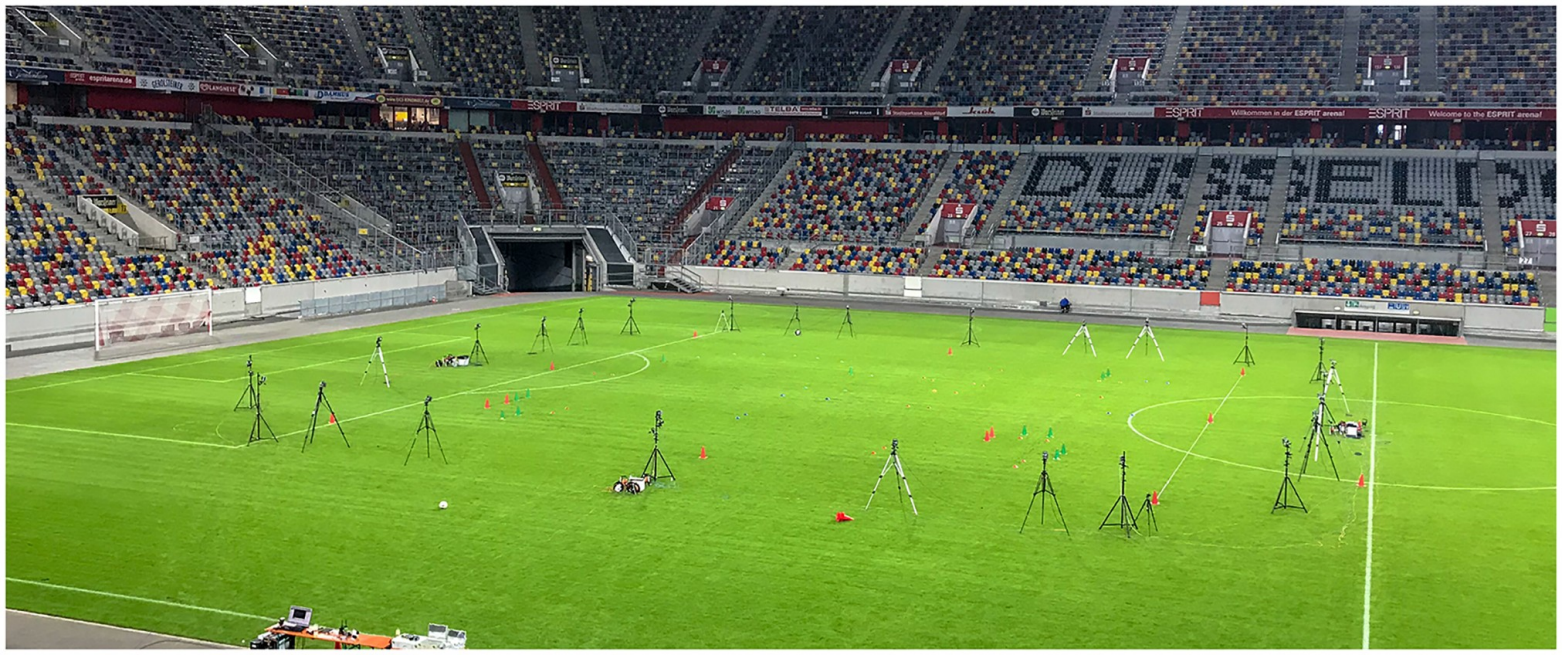
Image of the test setup at the ESPRIT Arena (Duesseldorf, Germany).

Measurements took place at the ESPRIT Arena (Duesseldorf, Germany, see Fig 1 for a visualisation of the test setup on-site). Twenty male soccer players (age: 22.9±3.6 years, height: 180.1±5.9 cm, body mass: 73.5±5.3 kg) playing for the German Mittelrheinliga team TSC Euskirchen participated in the study. Prior to participation, all players received comprehensive verbal and written explanations of the study, which was conducted within a period of two consecutive days. On each day, ten players participated. Players completed an individually selected warm-up before commencement of the tasks. Players were instructed to give maximal effort in all tasks. All players provided voluntarily signed informed consent to wear VICON markers and to participate in the collection of spatiotemporal tracking data. Institutional board approval for the study was obtained from the Ethics Committee of the Technical University of Munich. All performance data were anonymized to ensure confidentiality. This study conformed to the recommendations of the Declaration of Helsinki.

### Tested systems

**Fig 2:**
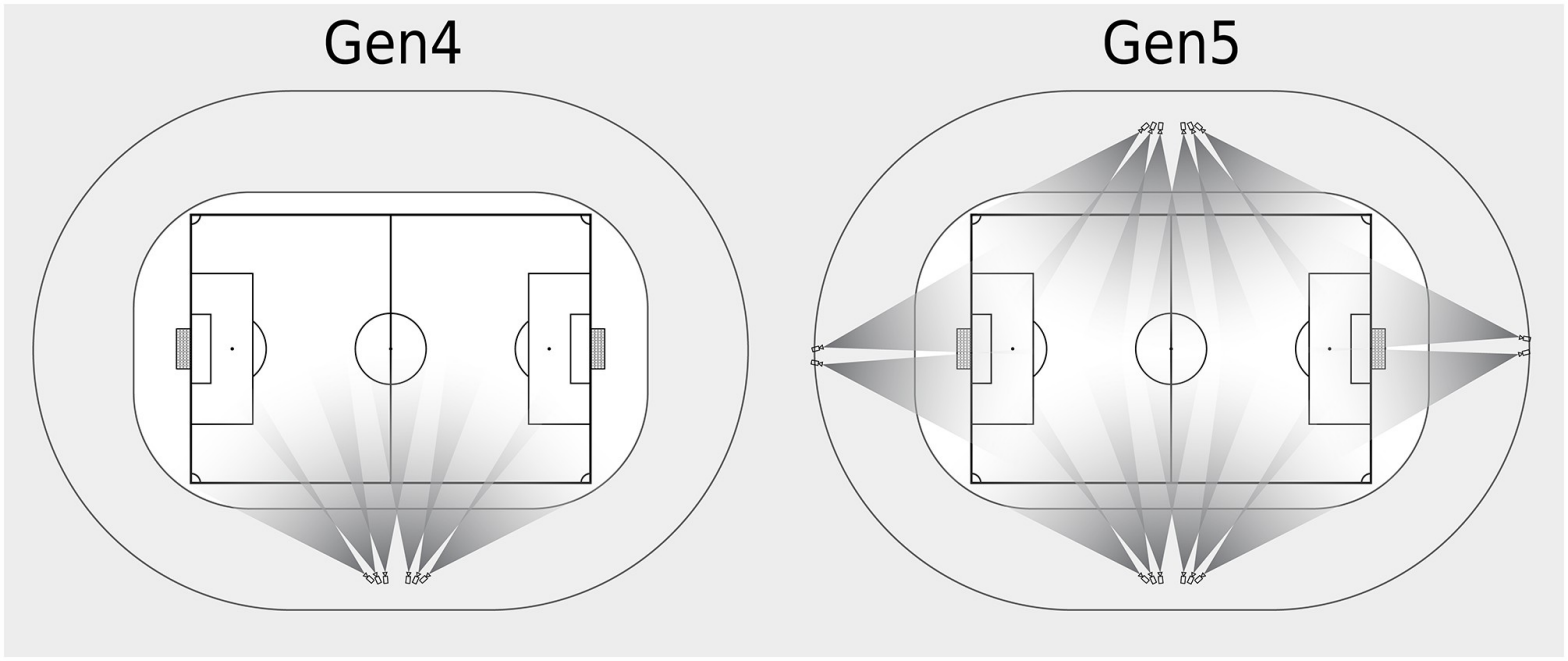
Spatial representation of the Gen4 (36m height) and Gen5(16m height) camera architecture.

Two different versions of TRACAB were validated: the 4^th^ generation TRACAB system (Gen4, installed at a height of 36m), and the 5^th^ generation (Gen5, installed at a height of 16m), which represents an advancement of the Gen4 system. The Gen4 system is a stereo camera system, consisting of two multi-camera units in two locations either side of the halfway line, each comprising three HD-SDI cameras with a resolution of 1920x1080 pixels (see Fig 2).

**Fig 3:**
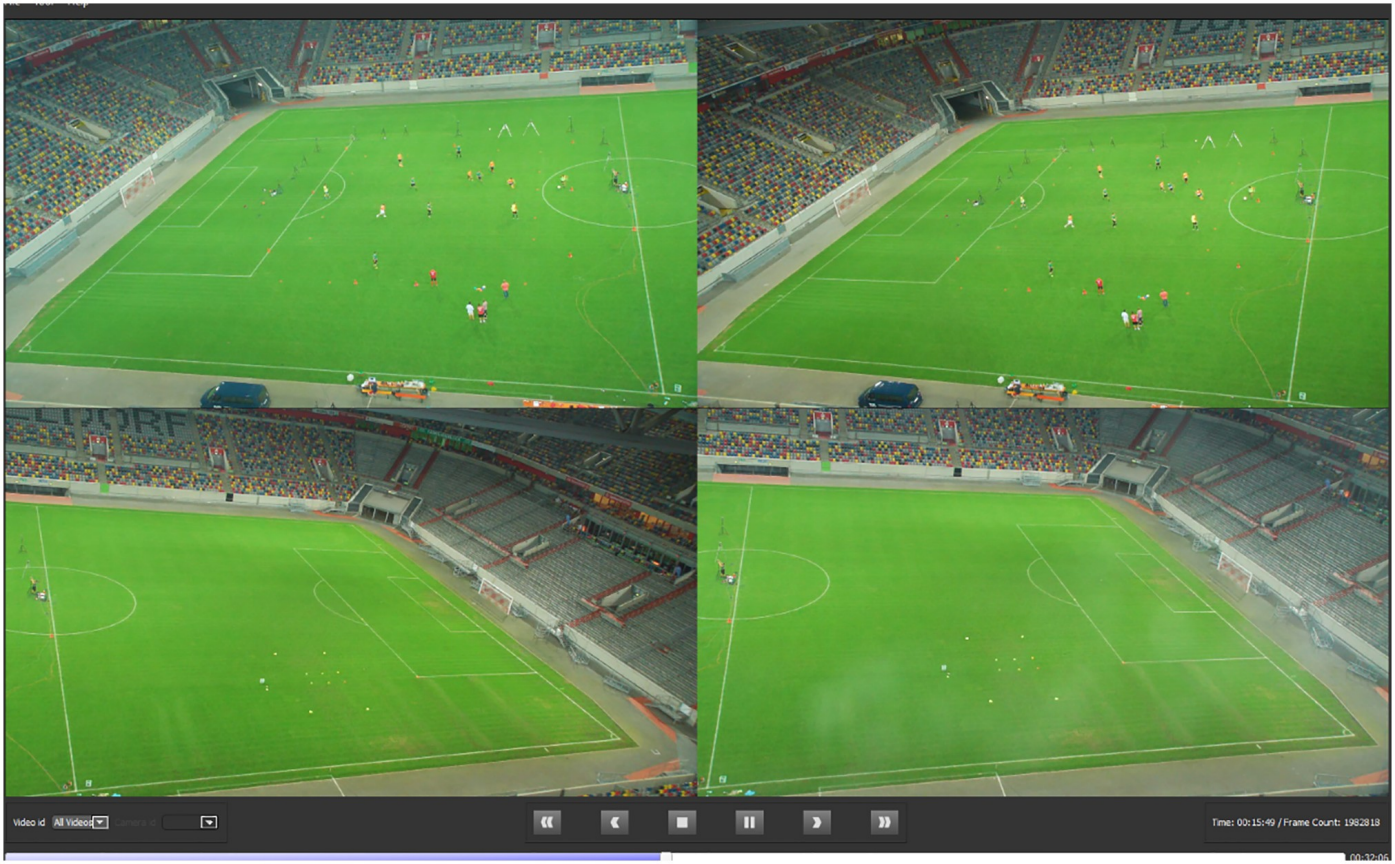
Exemplary still image of the camera setup at one of the four locations. The image shows four camera angles that are subsequently used to provide two panoramic pictures to be used as a stereo pair.

Each multi-camera unit provides a stitched panoramic picture, which is then used to create the stereoscopic view for triangulating the players and ball. The Gen5 system represents a distributed camera architecture, combining two stereo pairs on each side of the field as well as two monocular systems behind the goal areas. In total, the tested Gen5 systems comprised 16 IP-HD cameras with a resolution of 1920x1200 pixels. An exemplary still image of one of Gen5’s multiple-camera units is depicted in Fig 3.

It should be noted that optical tracking data is prone to errors introduced by occlusions and ID-swaps, which is why one human operator is required to correct these errors during measurement. For this reason, three types of tracking data can be distinguished: Type I data is considered ‘real-time’ data which is available immediately (<300ms), e.g. for live broadcasting, and therefore potentially contains several tracking errors. There is also Type II data, which is made available with approximately 15s delay and has less errors due to further processing and human interventions. Finally, Type III data describes the final data which is made available within several hours after the recordings. This final step comprises complete data correction and post-processing steps.

During our tests, the Gen4 system ran live (online) tracking during the recording session with a trained operator. The resulting Type II data was made available by ChyronHego immediately after the recording sessions. The Gen5 system recorded the same exercises. However, the video footage was later tracked offline using a real time tracking framework. When tracking the Gen5 footage, target identities were assigned at the beginning of each exercise. In all cases, the Gen5 system retained the correct target identities throughout the trial and no further operator interaction was required.

Accordingly, the data analysed in this study comprises exclusively Type II data, consisting of cartesian XY coordinates sampled with a frequency of 25 frames per second (25 Hz), which we from here on refer to as ‘raw’ tracking data, as no additional filtering methods or post-processing procedures have been applied.

### Criterion reference

Criterion reference data were collected by means of an infrared camera-based motion capture system (VICON, Oxford, UK, Software: Nexus, Version 2.3) at a sampling rate of 100 Hz. The setup used in this study consisted of 33 cameras mounted on tripods, evenly distributed around the observation field and calibrated using a custom-made rigid calibration object (see Fig 1). Retro-reflective markers with a diameter of 38.0 mm were used to assure stable recognition of the markers within the measurement area (30.0 x 30.0 m, 900.0 m^2^). In relation to the soccer field dimensions (105.0 x 68.0 m), the VICON measurement area was shifted towards the left half of the soccer field, flush with the 16-meter line of the penalty area (see Fig 1).

To demonstrate the spatial accuracy of the applied VICON setup, a rigid calibration object with precisely known dimensions was moved in increasing concentric circles to cover the entire measurement area. As the markers on the calibration object remain at accurately known distances to each other at any given time, the distances between the markers that are delivered by the VICON software, which are calculated in retrospect, can be used to document the measurement accuracy. Measurement errors in 3D space were estimated by means of the root mean square error (RMSE). In this study, the VICON setup achieved an accuracy of 1.16 mm root mean square error RMSE (95% CI [-2.09 mm, +2.17 mm]), thus meeting the requirements for a gold standard reference with errors being more than one magnitude smaller than the errors of the EPTS being tested [10].

**Fig 4:**
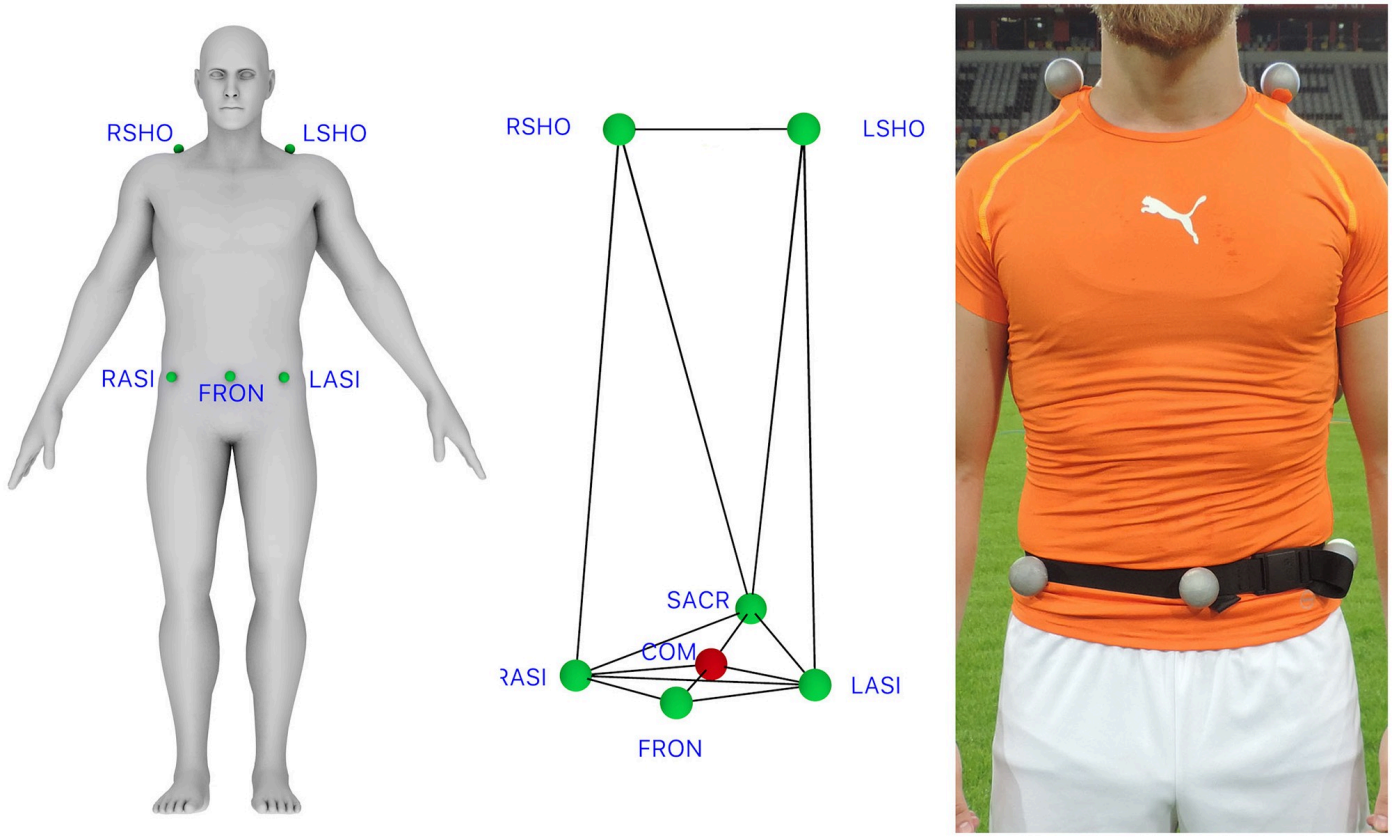
VICON marker positions on the human body (green), centre of mass COM (red).

On the assumption that EPTS endeavour to detect the position of the human body as a whole, the estimated centre of mass (COM) (or rather the XY-position of the body’s centre that is projected on the ground plane) was considered a valid criterion measure. To estimate COM, each player was equipped with six retro-reflective markers (right shoulder (RSHO), left shoulder (LSHO), left anterior superior iliac spine (LASI), right anterior superior iliac spine (RASI), sacrum (SACR), and front/umbilicus (FRON) (see Fig 4). COM was then estimated by means of the reconstructed pelvis method [29], defined as the geometric centre of RASI, LASI, SACR, and FRON.

### Exercises

**Fig 5:**
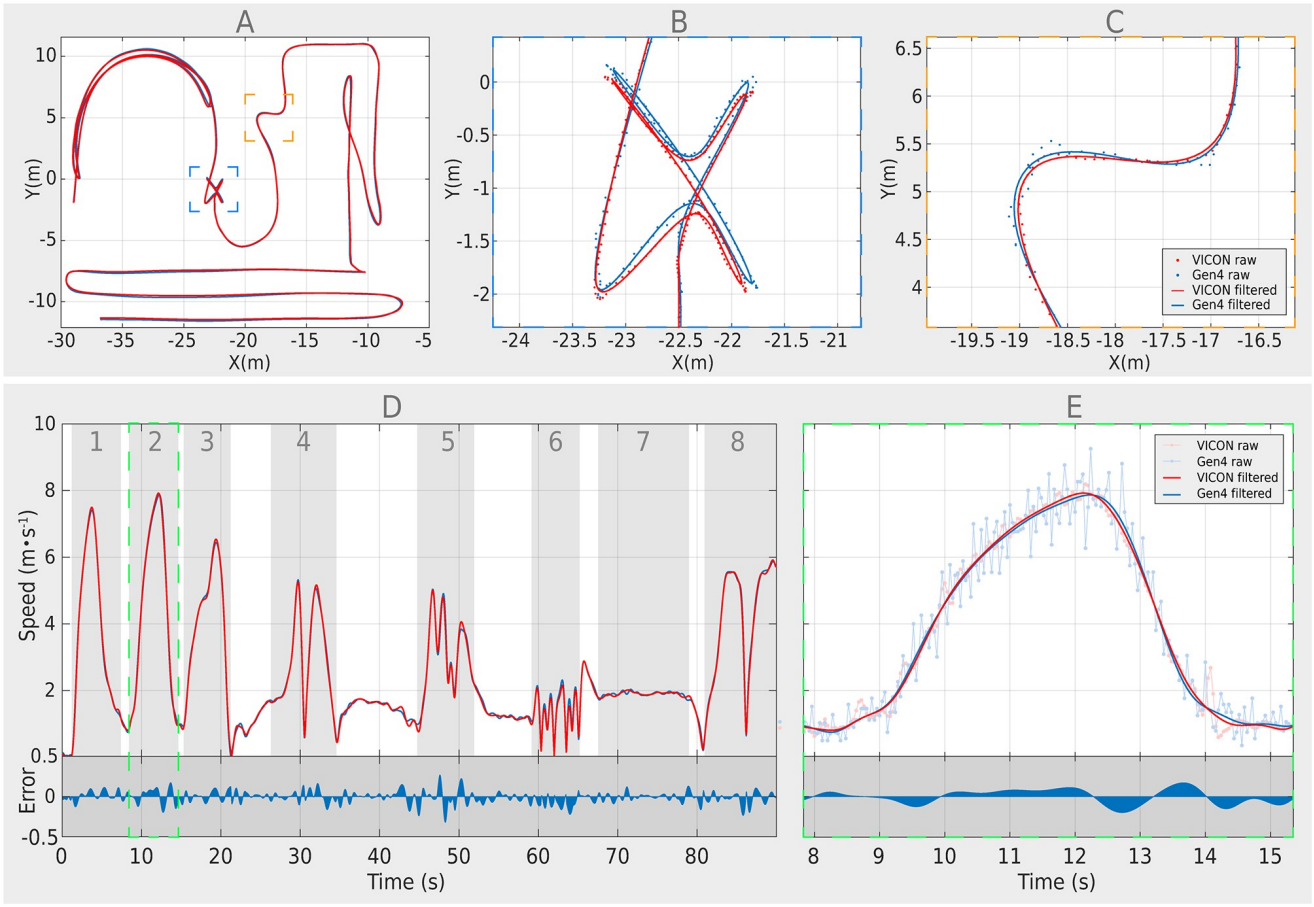
Exemplary illustration of both raw and smoothed position and speed data during a circuit. Subplot A shows the trajectories during a circuit. Subplot B and C show a magnification of two sections of the circuit (B = multidirectional lunges; C = sharp turns). Subplot D shows the derived speed including the individual sections 1–8. Subplot E shows a magnification of section2 (20m sprint).

A sport-specific running circuit with prescribed movement intensities was used to analyse elementary movements under controlled conditions and was similar to that which has been used previously to validate SportVU [28]. Within each trial, eight distinct elementary movement patterns were performed: (1) 15 m sprint into 5 m deceleration, (2) 20 m sprint, (3) 10 m backwards running into 10 m forward running, (4) 505 agility test, (5) rapid 90- & 135-degrees zig-zag turns, (6) multi-directional lunges, (7) slow curved runs, and (8) fast curved runs (see Fig 5). The beginning and end of each section were marked with two flat pylons, which in turn were equipped with reflective VICON markers. This enabled us in hindsight to detect the starting and endpoint of each section by means of the players’ XY-position (a player was located within/outside a certain section if his position crossed the line between the two start/end points).

5vs5 small- sided games were (SSG) played, without goals, as collective possession play. The format of the gameplay comprised of repeated 2-min bouts interspersed with 1-min of passive rest. Each drill was performed in a continuous regime, under the supervision, coaching, and motivation of the coaches to maintain a high work-rate. The ball was always available owing to prompt replacement any time it was hit out of the measurement area.

### Sample size

Table 1 summarizes the number of trials, recording time, and recorded data points (frames) included for analysis. VICON trials including data gaps >1 s were excluded from the analyses. This resulted in the exclusion of 5 SSC trials and 5 SSG trials from analysis, which resulted in a total number of 90 individual trials (a trial is defined as single observation of an individual player during an exercise).

**Table 1:** Sample size.

	Gen4	Gen5
Trials pooled	90	90
Trials SSG	55	55
Trials SSC	35	35
RecTime (min)	142.93	149.86
Frames pooled	214393	224792
Frames SSG	132382	148776
Frames SSC	82011	76016
Erroneous frames pooled	2336	0
Erroneous frames SSG	1426	0
Erroneous frames SSC	910	0

Only data points that were considered to be well tracked were evaluated, that is, where TRACAB’s position was within 1 meter of the associated ground truth position. The number of frames fulfilling this criterion is recorded in the “Frame” rows in the Table 1. Vice versa, frames not fulfilling this criterion were regarded as erroneous data and thus excluded from the analysis. Erroneous frames could, for example, result from unintentional ID swaps in situations where several players are standing closely together or where the systems falsely identified one of the coloured cones used to mark the circuit as a player.

1.08% of Gen4’s data points were found to be erroneous (resulting from 1.11% during SSCs and 1.07% during SSGs) (Table 1). No erroneous data points were detected in the analysis of the Gen5 system.

## Data processing

Estimating positional accuracy requires datasets to be precisely aligned. Therefore, datasets from VICON and TRACAB had to be aligned temporally and spatially. To achieve this, the original Vicon datasets were down-sampled from 100 Hz to 25 Hz to allow for comparisons with the TRACAB systems. The temporal alignment was then achieved by minimising the RMSE between the TRACAB and VICON signal, a method that provides a quantitative assessment of the similarity of the two signals at all possible time shifts, or time lags. The spatial alignment was achieved by means of a generalized Procrustes analysis (Euclidean similarity transformation, i.e. translation and rotation).

Data processing procedures, such as the derivation of kinematic metrics from position data, may vary drastically between different EPTS and even between different software versions of the same EPTS [27]. This issue is aggravated by the fact that manufacturer proprietary software often use data-processing algorithms that are subject to intellectual property protection, and their specific algorithms are not disclosed to the end user [27]. This complicates comparisons between different systems and software as different data processing decisions may lead to large deviations in the resulting kinematics [30]. In the context of EPTS validation studies it is, therefore, beneficial to apply the same data processing steps to both the tested system and the criterion reference. Otherwise, no conclusions can be drawn as to whether the observed deviations result from the system’s measurement inaccuracy or merely from differing data-processing decisions. In addition, the raw position might be the most unaffected variable and should be used as the primary variable to compare measurements between different positioning systems’ acquisition technology [21]. It is for this reason that we decided to apply the very same data processing steps to both VICON’s and TRACAB’s raw data.

Raw position data was filtered using a 4^th^ order low-pass Butterworth filter, a common filtering techniques in biomechanics [30]. This filter essentially passes signals with a frequency lower than a selected cut-off frequency and attenuates signals with frequencies higher than the cut-off frequency [31]. This method, however, requires the determination of a reasonable cut-off frequency, which will determine the amount of signal distortion and noise passed through the filter. Although the filtering of displacement signals to obtain noiseless velocities and accelerations has been widely studied in the literature, limited information exists on the appropriate cut-off frequencies of football-specific tracking data, nor is there any consensus on standardized filtering methods in previous research using comparable methodologies. The selection of a frequency cut-off is, however, a crucial part of the validation process as it has a significant impact on the resulting tracking variables, and thus also on the validation results.

To determine a reasonable cut-off frequency, we applied the method proposed by Winter [30], which essentially consists of performing a residual analysis to examine the differences between the raw and filtered position data over a pre-set range of cut-off frequencies (from 0.1 to 10 Hz in steps of 0.1 Hz). For both TRACAB and VICON, this method resulted in an optimal cut-off frequency between 1 and 1.5 Hz. For the purpose of this study, we took the conservative choice of rounding down and using a 1 Hz cut-off frequency on the raw position data. A visualisation of the raw and smoothed position data is illustrated in Fig 5.

### Kinematic and performance variables

Speed (scalar magnitude of velocity, as per rate of change in horizontal XY position) and acceleration (as per rate of change in speed) were derived from the filtered trajectories. Distance travelled for both VICON and TRACAB was calculated using the ‘waypoint method’, which attenuates the accumulation of small inconsequential movements and tracking inaccuracies that do not reflect the gross motion of a player [28]. Further, Key Performance Indicators (KPI, i.e. distance travelled while running with different ranges of speed, peak speed, peak acceleration, and peak deceleration) were calculated and utilised for the analysis. The minimum threshold for distance values to be considered in the analysis was 1 m. Running intensities were divided into the following speed thresholds: low speed (<6 km⋅h^-1^), moderate speed (>6 to <15 km⋅h^-1^), elevated speed (>15 to <20 km⋅h^-1^), high speed (>20 to <25 km⋅h^-1^), and very high speed (>25 km⋅h^-1^).

## Statistical analysis

For analysing position, speed and acceleration accuracy, the frames of all 90 trials were used as one dataset. Error indicators were calculated by using frames as statistical units and are presented in total, for each exercise and for each movement pattern by reporting the mean deviation and the root mean square errors (RMSE): pRMSE (m): position RMSE (horizontal distance between TRACAB and VICON); vRMSE (m⋅s^-1^): instant speed RMSE, and aRMSE (m⋅s^-2^): instant acceleration RMSE (Table 2). Percentiles PCTL_5/95_ are given as the 5^th^ and 95^th^ percentile (PCTL [5%, 95%]) (e.g., a 95^th^ percentile of 0.25 m means that 95% of measurements had an error of less than 0.25 m). A one-way between subjects analysis of variance (ANOVA) was conducted to test if the mean error between the two tested systems differs. Accuracy analysis of KPIs used individual trials as statistical units. Accuracy analysis of KPIs used individual trials as statistical units. KPIs and KPI differences between systems are reported as mean ± SD for each system (Table 3). Two-tailed paired t-tests were used to compare the KPIs derived by TRACAB Gen4 and Gen5 with that derived from VICON. In the same way, TRACAB Gen4 data was compared with Gen5 data. KPI deviations between two systems are reported as the KPI differences in percentages of the first system’s KPI (Table 4).

**Table 2:** Validation results of position, speed and acceleration accuracy, presented as root mean square error (RMSE), mean, and 5^th^ and 95^th^ percentiles.

	Exercise	Gen4 vs VICON		Gen5 vs VICON		Gen4 vs Gen5		
		RMSE	Mean I PCTL	RMSE	Mean I PCTL	Test Statistic	ES	
**pRMSE (m)**	Pooled	0.09	0.08 [0.01, 0.18]	0.08	0.07 [0.02, 0.15]	[F(1, 399170) = 162.278, p < 0.001]	0.04	*trivial*
	Circuit	0.09	0.08 [0.02, 0.16]	0.09	0.08 [0.02, 0.15]	[F(1, 145880) = 200.823, p < 0.001]	0.07	*trivial*
	Small sided game	0.09	0.07 [0.01, 0.19]	0.08	0.07 [0.02, 0.14]	[F(1, 253288) = 34.133, p < 0.001]	0.02	*trivial*
	Low speed location change	0.07	0.06 [0.01, 0.13]	0.08	0.07 [0.02, 0.14]	[F(1, 63467) = 81.432, p < 0.001]	0.07	*trivial*
	15m sprint / 5m deceleration	0.18	0.14 [0.04, 0.38]	0.10	0.09 [0.02, 0.18]	[F(1, 2377) = 318.138, p < 0.001]	0.72	*moderate*
	20 sprint	0.12	0.11 [0.03, 0.20]	0.10	0.09 [0.02, 0.17]	[F(1, 6374) = 298.290, p < 0.001]	0.43	*small*
	10m backwards / 10m forward	0.10	0.09 [0.02, 0.18]	0.08	0.07 [0.02, 0.13]	[F(1, 8882) = 226.632, p < 0.001]	0.32	*small*
	505 agility test	0.08	0.08 [0.02, 0.14]	0.09	0.08 [0.02, 0.15]	[F(1, 11524) = 16.918, p < 0.001]	0.08	*trivial*
	Sharp turns 90 & 135	0.08	0.07 [0.02, 0.15]	0.12	0.11 [0.03, 0.20]	[F(1, 12032) = 1636.890, p < 0.001]	0.74	*moderate*
	Lunges	0.07	0.06 [0.02, 0.10]	0.08	0.08 [0.02, 0.14]	[F(1, 8091) = 408.983 p < 0.001]	0.45	*small*
	Curved run slow	0.11	0.10 [0.03, 0.19]	0.07	0.06 [0.02, 0.12]	[F(1, 18915) = 3065.512, p < 0.001]	0.82	*large*
	Curved run fast	0.12	0.10 [0.03, 0.20]	0.11	0.10 [0.03, 0.17]	[F(1, 14202) = 80.852, p < 0.001]	0.15	*trivial*
**vRMSE (m/s)**	Pooled	0.09	-0.01 [-0.11, 0.08]	0.08	-0.01 [-0.10, 0.09]	[F(1, 399170) = 755.156, p < 0.001]	0.06	*trivial*
	Circuit	0.06	-0.01 [-0.10, 0.08]	0.05	-0.00 [-0.09, 0.08]	[F(1, 145880) = 496.685, p < 0.001]	0.04	*trivial*
	Small sided game	0.10	-0.02 [-0.12, 0.08]	0.09	-0.01 [-0.10, 0.09]	[F(1, 253288) = 396.649, p < 0.001]	0.07	*trivial*
	Low speed location change	0.04	-0.00 [-0.07, 0.06]	0.05	0.00 [-0.07, 0.07]	[F(1, 63467) = 80.906, p < 0.001]	0.03	*trivial*
	15m sprint / 5m deceleration	0.13	0.03 [-0.17, 0.27]	0.09	0.01 [-0.16, 0.11]	[F(1, 2377) = 19.554, p < 0.001]	0.22	*trivial*
	20 sprint	0.13	-0.02 [-0.26, 0.12]	0.06	0.01 [-0.11, 0.10]	[F(1, 6374) = 79.053, p < 0.001]	0.48	*small*
	10m backwards / 10m forward	0.05	-0.02 [-0.09, 0.08]	0.06	-0.00 [-0.10, 0.09]	[F(1, 8882) = 81.442, p < 0.001]	0.07	*trivial*
	505 agility test	0.06	0.00 [-0.10, 0.11]	0.06	0.00 [-0.10, 0.09]	[F(1, 11524) = 2.999, p = 0.083]	0.10	*trivial*
	Sharp turns 90 & 135	0.07	-0.02 [-0.12, 0.09]	0.07	-0.00 [-0.12, 0.12]	[F(1, 12032) = 149.321, p < 0.001]	0.11	*trivial*
	Lunges	0.05	-0.02 [-0.11, 0.06]	0.04	-0.01 [-0.08, 0.05]	[F(1, 8091) = 24.414, p < 0.001]	0.22	*small*
	Curved run slow	0.03	-0.01 [-0.06, 0.04]	0.03	-0.00 [-0.05, 0.04]	[F(1, 18915) = 414.430, p < 0.001]	0.14	*trivial*
	Curved run fast	0.09	-0.03 [-0.14, 0.08]	0.08	-0.02 [-0.14, 0.10]	[F(1, 14202) = 102.515, p < 0.001]	0.03	*trivial*
**aRMSE (m/s2)**	Pooled	0.26	-0.00 [-0.23, 0.24]	0.21	-0.00 [-0.21, 0.21]	[F(1, 399170) = 2.102, p = 0.147]	0.08	*trivial*
	Circuit	0.22	-0.00 [-0.20, 0.22]	0.16	0.00 [-0.19, 0.20]	[F(1, 145880) = 0.290, p = 0.590]	0.05	*trivial*
	Small sided game	0.27	-0.00 [-0.25, 0.26]	0.23	-0.00 [-0.22, 0.22]	[F(1, 253288) = 1.857, p = 0.173]	0.09	*trivial*
	Low speed location change	0.14	-0.01 [-0.16, 0.14]	0.13	-0.00 [-0.14, 0.15]	[F(1, 63467) = 19.763, p < 0.001]	0.01	*trivial*
	15m sprint / 5m deceleration	0.68	0.17 [-0.60, 1.69]	0.25	-0.02 [-0.43, 0.32]	[F(1, 2377) = 93.501, p < 0.001]	0.44	*small*
	20 sprint	0.63	-0.08 [-1.08, 0.51]	0.13	-0.00 [-0.23, 0.19]	[F(1, 6374) = 42.837, p < 0.001]	0.48	*small*
	10m backwards / 10m forward	0.12	0.00 [-0.18, 0.20]	0.13	-0.01 [-0.21, 0.20]	[F(1, 8882) = 15.098, p < 0.001]	0.18	*trivial*
	505 agility test	0.26	0.01 [-0.16, 0.26]	0.27	0.00 [-0.17, 0.25]	[F(1, 11524) = 0.207, p = 0.649]	0.04	*trivial*
	Sharp turns 90 & 135	0.19	0.00 [-0.31, 0.30]	0.19	-0.00 [-0.28, 0.31]	[F(1, 12032) = 0.054, p = 0.816]	0.07	*trivial*
	Lunges	0.19	0.01 [-0.23, 0.29]	0.15	0.01 [-0.20, 0.25]	[F(1, 8091) = 0.172, p = 0.678]	0.11	*trivial*
	Curved run slow	0.06	0.00 [-0.08, 0.08]	0.06	-0.00 [-0.09, 0.08]	[F(1, 18915) = 7.082, p = 0.008]	0.06	*trivial*
	Curved run fast	0.24	0.02 [-0.25, 0.30]	0.25	0.01 [-0.24, 0.29]	[F(1, 14202) = 0.131, p = 0.717]	0.03	*trivial*

**Table 3:** Means of the deviation in total distance, distance travelled while running with different ranges of speed, peak speed, peak acceleration, and peak deceleration between VICON and Gen4, VICON and Gen5, and Gen4 and Gen5. The tested variables each comprise 90 paired values of the respective tracking variable (one for each trial).

	KPI	VICON	Gen4	Gen5
		Mean ± SD	Mean ± SD	Mean ± SD
**KPI means**	Total distance (m)	205.16 ± 19.40	206.03 ± 19.60	205.71 ± 19.45
	Low speed distance (m)	44.53 ± 11.72	37.60 ± 10.28	43.73 ± 11.45
	Moderate speed distance (m)	112.04 ± 40.65	117.17 ± 41.34	113.02 ± 41.50
	Elevated speed distance (m)	29.55 ± 15.60	31.19 ± 15.30	29.33 ± 15.55
	High speed distance (m)	31.85 ± 11.49	34.58 ± 11.80	33.10 ± 11.53
	Sprinting distance (m)	15.61 ± 5.25	14.71 ± 5.24	15.75 ± 5.34
	Peak speed (m/s)	6.36 ± 1.71	6.43 ± 1.71	6.36 ± 1.70
	Peak acceleration (m/s2)	7.21 ± 3.64	7.22 ± 3.62	7.19 ± 3.59
	Peak deceleration (m/s2)	-6.97 ± 3.75	-7.01 ± 3.74	-6.99 ± 3.73

**Table 4:** Test statistics of the deviation in total distance, distance travelled while running with different ranges of speed, peak speed, peak acceleration, and peak deceleration between VICON and Gen4, VICON and Gen5, and Gen4 and Gen5. The tested variables each comprise 90 paired values of the respective tracking variable (one for each trial).

	KPI	Gen4 vs VICON				Gen5 vs VICON				Gen4 vs Gen5			
		% Diff	p	ES		% Diff	p	ES		% Diff	p	ES	
**Test statistics**	Total distance (m)	0.42 ± 0.60	0.00	0.04	*trivial*	0.27 ± 0.35	0.00	0.03	*trivial*	-0.15 ± 0.37	0.00	0.02	*trivial*
	Low speed distance (m)	-19.41 ± 13.24	0.00	0.63	*moderate*	-2.02 ± 4.15	0.00	0.07	*trivial*	13.77 ± 7.69	0.00	0.53	*moderate*
	Moderate speed distance (m)	4.68 ± 5.06	0.00	0.13	*trivial*	0.61 ± 2.12	0.00	0.02	*trivial*	-4.52 ± 5.60	0.00	0.10	*trivial*
	Elevated speed distance (m)	7.52 ± 10.08	0.00	0.11	*trivial*	-1.58 ± 6.76	0.22	0.01	*trivial*	-11.11 ± 14.08	0.00	0.12	*trivial*
	High speed distance (m)	8.94 ± 9.49	0.00	0.23	*small*	4.98 ± 8.79	0.00	0.12	*trivial*	-5.49 ± 5.94	0.00	0.13	*trivial*
	Sprinting distance (m)	-9.13 ± 25.58	0.03	0.17	*trivial*	-0.49 ± 7.01	0.86	0.01	*trivial*	5.94 ± 13.25	0.01	0.20	*small*
	Peak speed (m/s)	1.13 ± 1.97	0.00	0.04	*trivial*	0.14 ± 1.34	0.41	0.00	*trivial*	-1.08 ± 1.81	0.00	0.04	*trivial*
	Peak acceleration (m/s2)	-0.13 ± 5.25	0.82	0.00	*trivial*	-0.33 ± 5.09	0.98	0.00	*trivial*	-0.39 ± 3.05	0.24	0.01	*trivial*
	Peak deceleration (m/s2)	0.49 ± 5.27	0.10	0.01	*trivial*	0.42 ± 4.87	0.38	0.01	*trivial*	-0.22 ± 2.50	0.22	0.01	*trivial*

Effect sizes (ES) were quantified to indicate the meaningfulness of the differences in the mean values. Cohen’s d effect sizes were classified as *trivial* (0–0.19), *small* (0.20–0.49), *moderate* (0.50–0.79) and *large* (>0.80) [32]. Holm-Bonferroni method was applied to counteract the problem of multiple comparisons. Before using parametric statistical test procedures, the assumption of normality was verified. Statistical significance was set at p < 0.05. All data analyses were conducted in MATLAB (Release 2018b, The MathWorks, Inc., USA).

## Results

### Accuracy of spatiotemporal tracking data

Deviations of momentary position, speed and acceleration between TRACAB and VICON are presented in Table 2. In total, Gen5 (0.08 m pRMSE) errors indicators for position estimations were smaller compared to Gen4 (0.09 m pRMSE), but the effect size was trivial. *Moderate* differences were observed during 15m sprints (Gen4: 0.18 m pRMSE; Gen5: 0.1 m pRMSE) and sharp turns (Gen4: 0.08 m pRMSE; Gen5: 0.12 m pRMSE), and *large* differences during slow curved runs (Gen4: 0.11 m pRMSE; Gen5: 0.07 m pRMSE).

The accuracy difference in instantaneous speed measurements across all exercises was also significant, but *trivial* (Gen4: 0.09 m⋅s^-1^ vRMSE; Gen5: 0.08 m⋅s^-1^ vRMSE). *Small* differences in instantaneous speed accuracy were found during 20m sprints (Gen4: 0.13 m⋅s^-1^ vRMSE; Gen5: 0.06 m⋅s^-1^ vRMSE) and lunges (Gen4: 0.05 m⋅s^-1^ vRMSE; Gen5: 0.04 m⋅s^-1^ vRMSE).

Similarly, the accuracy difference in instantaneous acceleration measurements across all exercises was significant, but *trivial* (Gen4: 0.26 m⋅s^-2^ aRMSE; Gen5: 0.21 m⋅s^-2^ aRMSE). *Small* differences in instantaneous acceleration accuracy were found during 15m sprints (Gen4: 0.68 m⋅s^-2^ aRMSE; Gen5: 0.25 m⋅s^-2^ aRMSE) and 20m sprints (Gen4: 0.63 m⋅s^-2^ aRMSE; Gen5: 0.13 m⋅s^-2^ aRMSE).

### Results of key performance indicators (KPI)

Deviation in KPIs between TRACAB and VICON are presented in Tables 3 & 4.

For total distance travelled, both Gen4 (0.42 ± 0.60%) and Gen5 (0.27 ± 0.35%) showed only *trivial* differences compared to VICON. Whereas Gen5 showed only trivial differences in all tested KPI categories, Gen4 showed *moderate* differences in the low speed distance travelled category (-19.41 ± 13.24%) and *small* differences in the high-speed distance travelled category (8.94 ± 9.49%). Differences in peak speed, acceleration, and deceleration were trivial for both Gen4 and Gen5.

## Discussion

### Discussion of results

The current study aimed to validate and compare the spatiotemporal measurement accuracy of TRACAB’s optical player tracking systems Gen4 and Gen5 against a criterion reference under football-specific test conditions. Overall, we found that Gen5 had better accuracy for measuring a player’s momentary position than Gen4. The observed difference in positional accuracy was statistically significant but *trivial* in terms of the magnitude. Accordingly, analysis of instantaneous speed and acceleration accuracy showed a similar picture, with Gen5’s deviations being *trivially* smaller than the deviations of Gen4. Considering KPIs, only *trivial* differences were found between performance indicators derived from VICON and Gen5, while Gen4 showed *small-to-moderate* differences in distances travelled with low and high speeds.

Compared with previous EPTS research that analysed the position accuracy under dynamic test conditions, it is apparent that both Gen4’s and Gen5’s position errors (7–8 cm mean absolute error; 8–9 cm pRMSE) appear to be considerably smaller than the errors of previously tested video tracking systems (56 cm [28]), as well as radio-based LPS systems (19–27 cm [8, 28][21]). Several factors could explain this notable increase in positioning accuracy: first and foremost, it is well documented that the spatial accuracy and robustness of optical tracking systems are primarily dependent on the number, resolution, distribution, and viewing angle of the utilised cameras [4]. Both Gen4 (six cameras with 1920x1080 pixels) and Gen5 (16 cameras with 1920x1200 pixels) had a considerably higher combined resolution than the previously tested video tracking system, which utilised only three cameras with a resolution of 2560x1500 pixel [28].

In addition to the number and resolution of utilised cameras, a key differentiator between optical tracking systems is the distribution of cameras around the pitch and, associated therewith, whether the system is considered a “single-view” system (as is the case with Gen4) or a “multi-view” or “distributed” camera system (as is the case with Gen5 in which several camera units are installed on all four sides of the pitch). Using a distributed camera system has the obvious advantage that it enables recordings from different perspectives, thus reducing the number of occlusions and possibly having a higher pixel resolution especially on small distant players [4, 33]. Despite these advantages, distributed camera systems introduce further complications such as difficulties in the camera setup, robustness in configuration, collaboration among multiple views, increased hardware costs, and increased computational complexity, which makes them impractical and relatively expensive for real-time applications [4, 34]. Notwithstanding these limitations, there are reasons to consider that, by now, ongoing improvements in processing power and tracking algorithms justify the implementation of distributed camera systems.

Despite the higher number of cameras (6 vs 16), we found only *trivial* differences (1 cm) in positioning accuracy between Gen4 and Gen5. It should, however, be noted that 1% of Gen4’s tracking data had to be excluded from analysis due to tracking errors such as gaps in the data or ID-swaps. The majority of Gen4’s tracking errors occurred during the first section of the circuit (15m sprint), caused by tracking inaccuracies induced by players passing the coloured cones that were needed to mark the circuits’ running paths. Accordingly, Gen4’s position errors were significantly larger during this specific section. None of these tracking inaccuracies was found in Gen5’s data (0% tracking errors), suggesting that additional perspectives decrease the number of occlusions and tracking errors caused by the insufficient image quality.

In the case of total distance travelled, the mean bias compared to VICON was <0.5% for both Gen4 and Gen5, which is well in line with values of 0.1–3.5% reported by previous investigations utilizing several types of GPS-, radio-, and video-based EPTS [20, 21, 23, 35–37]. Likewise, we found only *trivial* differences in peak speed measures for both Gen4 (1.13%) and Gen5 (0.14%), which is comparable with recent validations of four different GNSS and LPS systems (<2.1%, [38, 39]. Collectively, these results confirm once again that today’s state-of-the-art EPTS are generally capable of measuring the total distance travelled and peak speed with sufficient accuracy—regardless of the underlying technology.

While total distance is generally used as a proxy of overall training volume, high-intensity distance and acceleration/deceleration patterns are believed to be the most important variables to be tracked since they refer to a more neuromuscular-oriented type of load, which is more likely to be linked with injury risk [1, 40]. Unfortunately, however, previous research has demonstrated that the validity and reliability of high-intensity performance indicators is likely inversely related to their importance in terms of load monitoring, that is, high-speed running, acceleration/deceleration work, and metabolic power being the least valid and reliable variables [40, 41]. Recent examples indeed showed deviations of 20–40% in distances travelled while running with high intensities [28, 42], and up to 41% for peak decelerations during walking, and 8.9% during changes of direction [35]. In contrast to that, this study found only *trivial* biases in peak acceleration and deceleration values for both Gen4 and Gen5 (<0.5%) and *small-to-trivial* (Gen4, <10%) and *trivial* (Gen5, <5%) deviations in high-intensity distance metrics (comprising high-speed running and sprinting distance). It is worth mentioning, however, that a previous VICON validation of Inmotio’s LPM likewise found equally trivial biases for peak acceleration and deceleration values (<0.5% [43]). This large margin in the reported deviations, particularly in peak acceleration and deceleration values, demonstrates once more that comparison between different studies should only be with caution and only in consideration of the underlying data processing decisions.

### Discussion of methods

As is the case with every EPTS validation study, our results should only be interpreted in consideration of the applied data processing procedures and in particular the applied data smoothing method. In this context, it should be emphasised that we deliberately chose to apply the very same data processing and smoothing methods to the raw positional data of both the criterion reference and the tested systems. This is the only way to ensure that the observed deviations are not caused by arbitrary differences in data processing decisions, but in fact by the fundamental data capturing capability of the tested tracking technology.

Particular caution is advised in the direct comparison of peak acceleration and deceleration results between different studies as the magnitude of instantaneous acceleration values is significantly influenced by the applied data smoothing decisions. Due to the necessary step of differentiation, minor differences in the smoothing procedures applied to the raw positional data can lead to exponentially rising deviations in instantaneous speed and acceleration values. We should not be surprised, therefore, that the vast majority of previously tested EPTS seems capable of measuring the most fundamental performance indicators (e.g., total distance travelled, peak speed) with reasonable accuracy, whereas we tend to find drastically increasing deviations in high-intensity performance indicators. Instead, future research should aim at defining reasonable standards for EPTS specific data processing methods in order to reduce the influence of different data processing decisions on the resulting validation results.

Our results are limited by the fact that the tracking systems were tested in a professional football stadium with a retractable roof and, therefore, under favourable conditions. This particular infrastructure made it possible to install the systems at optimal height and distance from the field and enabled data recordings under consistent weather and lighting conditions.

Our tests did not include the most challenging situations for optical tracking systems (e.g., corners, free kicks) where many tightly packed players lead to tracking errors and ID-swaps as the players re-disperse. Therefore, it should be noted that the reported percentage values of tracking errors are not representative of real match scenarios.

## Conclusions

Based on the present results, we conclude that both Gen4 and Gen5 can be considered as valid technologies for football-specific performance analyses in the settings tested providing players are tracked correctly. Our results showed significant but trivial differences in the accuracy of momentary position, speed and acceleration measures between Gen4 and Gen5. When considering typical performance indicators, we also found only trivial differences in KPIs derived from VICON and Gen5, whereas Gen4 showed small-to-moderate differences in distances travelled with low and high speeds compared to the two other systems. There are indications that Gen5’s distributed camera architecture has minor benefits over Gen4’s single-view camera architecture when it comes to robustness of tracking.

## Supporting information

S1 Data (CSV)

## References

[1] Buchheit M, Simpson BM. Player-Tracking Technology: Half-Full or Half-Empty Glass? Int J Sports Physiol Perform. 2017;12(Suppl 2):S2-35–S2-41. 10.1123/ijspp.2016-0499 27967285

[2] Memmert D, Raabe D. Data Analytics in Football: Positional Data Collection, Modelling and Analysis: Routledge; 2018.

[3] Alamar BC. Sports analytics: A guide for coaches, managers, and other decision makers: Columbia University Press; 2013.

[4] Manafifard M, Ebadi H, Moghaddam HA. A survey on player tracking in soccer videos. Computer Vision and Image Understanding. 2017.

[5] Oskouie P, Alipour S, Eftekhari-Moghadam A-M. Multimodal feature extraction and fusion for semantic mining of soccer video: a survey. Artificial Intelligence Review. 2014;42(2):173–210.

[6] Castellano J, Alvarez-Pastor D, Bradley PS. Evaluation of research using computerised tracking systems (Amisco and Prozone) to analyse physical performance in elite soccer: a systematic review. Sports Med. 2014;44(5):701–12. 10.1007/s40279-014-0144-3 24510701

[7] Scott MT, Scott TJ, Kelly VG. The validity and Reliability of Global Positioning Systems In Team Sport: A Brief Review. The Journal of Strength & Conditioning Research. 2016;30(5):1470–90. 26439776 10.1519/JSC.0000000000001221

[8] Ogris G, Leser R, Horsak B, Kornfeind P, Heller M, Baca A. Accuracy of the LPM tracking system considering dynamic position changes. J Sports Sci. 2012;30(14):1503–11. 10.1080/02640414.2012.712712 22906154

[9] Link D, Hoernig M. Individual ball possession in soccer. PLoS One. 2017;12(7):e0179953 10.1371/journal.pone.0179953 28692649 PMC5503225

[10] Linke D, Link D, Weber H, Lames M. Decline in Match Running Performance in Football is affected by an Increase in Game Interruptions. J Sports Sci Med. 2018;17(4):662 30479536 PMC6243615

[11] Castellano J, Casamichana D. What are the differences between first and second divisions of Spanish football teams? International Journal of Performance Analysis in Sport. 2015;15(1):135–46.

[12] Castellano J, Blanco-Villaseñor A. Analysis of the variability of the movement of elite soccer players during a competitive season of a generalized linear mixed model. Cuadernos de Psicología del Deporte. 2015;15(1):161–8.

[13] Andrienko G, Andrienko N, Budziak G, Dykes J, Fuchs G, von Landesberger T, et al Visual analysis of pressure in football. Data Mining and Knowledge Discovery. 2017;31(6):1793–839.

[14] Dick U, Brefeld U. Learning to Rate Player Positioning in Soccer. Big data. 2019;7(1):71–82. 10.1089/big.2018.0054 30672712

[15] Link D, Lang S, Seidenschwarz P. Real time quantification of dangerousity in football using spatiotemporal tracking data. PLoS One. 2016;11(12):e0168768 10.1371/journal.pone.0168768 28036407 PMC5201291

[16] Memmert D, Lemmink KA, Sampaio J. Current approaches to tactical performance analyses in soccer using position data. Sports Med. 2017;47(1):1–10. 10.1007/s40279-016-0562-5 27251334

[17] Link D, Kolbinger O, Weber H, Stöckl M. A topography of free kicks in soccer. J Sports Sci. 2016;34(24):2312–20. 10.1080/02640414.2016.1232487 27892396

[18] Bastida Castillo A, Gómez Carmona CD, De la cruz sánchez E, Pino Ortega J. Accuracy, intra-and inter-unit reliability, and comparison between GPS and UWB-based position-tracking systems used for time–motion analyses in soccer. European journal of sport science. 2018;18(4):450–7. 29385963 10.1080/17461391.2018.1427796

[19] Beato M, Bartolini D, Ghia G, Zamparo P. Accuracy of a 10 Hz GPS Unit in Measuring Shuttle Velocity Performed at Different Speeds and Distances (5–20 M). Journal of Human Kinetics. 2016;54(1):15–22. 28031753 10.1515/hukin-2016-0031 PMC5187957

[20] Frencken WG, Lemmink KA, Delleman NJ. Soccer-specific accuracy and validity of the local position measurement (LPM) system. J Sci Med Sport. 2010;13(6):641–5. 10.1016/j.jsams.2010.04.003 20594910

[21] Luteberget LS, Spencer M, Gilgien M. Validity of the Catapult ClearSky T6 local positioning system for team sports specific drills, in indoor conditions. Front Physiol. 2018;9:115 10.3389/fphys.2018.00115 29670530 PMC5893723

[22] Vickery WM, Dascombe BJ, Baker JD, Higham DG, Spratford WA, Duffield R. Accuracy and reliability of GPS devices for measurement of sports-specific movement patterns related to cricket, tennis, and field-based team sports. The Journal of Strength & Conditioning Research. 2014;28(6):1697–705. 24149747 10.1519/JSC.0000000000000285

[23] Di Salvo V, Collins A, McNeill B, Cardinale M. Validation of Prozone: a new video-based performance analysis system. International Journal of Performance Analysis in Sport. 2006;6(1):108–19.

[24] Buchheit M, Allen A, Poon TK, Modonutti M, Gregson W, Di Salvo V. Integrating different tracking systems in football: multiple camera semi-automatic system, local position measurement and GPS technologies. J Sports Sci. 2014;32(20):1844–57. 10.1080/02640414.2014.942687 25093242

[25] Redwood-Brown A, Cranton W, Sunderland C. Validation of a Real-Time Video Analysis System for Soccer. Int J Sports Med. 2012;33(8):635–40. 10.1055/s-0032-1306326 22510800

[26] Aughey RJ. Applications of GPS technologies to field sports. Int J Sports Physiol Perform. 2011;6(3):295–310. 10.1123/ijspp.6.3.295 21911856

[27] Malone JJ, Lovell R, Varley MC, Coutts AJ. Unpacking the black box: applications and considerations for using GPS devices in sport. Int J Sports Physiol Perform. 2017;12(Suppl 2):S2-18–S2-26. 10.1123/ijspp.2016-0236 27736244

[28] Linke D, Link D, Lames M. Validation of electronic performance and tracking systems EPTS under field conditions. PLoS One. 2018;13(7):e0199519 10.1371/journal.pone.0199519 30036364 PMC6056042

[29] Saini M, Kerrigan D, Thirunarayan M, Duff-Raffaele M. The vertical displacement of the center of mass during walking: a comparison of four measurement methods. J Biomech Eng. 1998;120(1):133–9. 10.1115/1.2834293 9675692

[30] Winter DA. Biomechanics and motor control of human movement: John Wiley & Sons; 2009.

[31] Sinclair J, Taylor PJ, Hobbs SJ. Digital filtering of three-dimensional lower extremity kinematics: An assessment. Journal of human kinetics. 2013;39(1):25–36. 24511338 10.2478/hukin-2013-0065 PMC3916920

[32] Cohen J. A power primer. Psychol Bull. 1992;112(1):155 10.1037//0033-2909.112.1.155 19565683

[33] Javed O, Rasheed Z, Shafique K, Shah M, editors. Tracking across multiple cameras with disjoint views. Proceedings of the Ninth IEEE International Conference on Computer Vision-Volume 2; 2003: IEEE Computer Society.

[34] Baysal S, Duygulu P. Sentioscope: a soccer player tracking system using model field particles. IEEE Transactions on Circuits and Systems for Video Technology. 2016;26(7):1350–62.

[35] Serpiello F, Hopkins W, Barnes S, Tavrou J, Duthie G, Aughey R, et al Validity of an ultra-wideband local positioning system to measure locomotion in indoor sports. J Sports Sci. 2018;36(15):1727–33. 10.1080/02640414.2017.1411867 29192842

[36] Johnston RJ, Watsford ML, Kelly SJ, Pine MJ, Spurrs RW. Validity and interunit reliability of 10 Hz and 15 Hz GPS units for assessing athlete movement demands. The Journal of Strength & Conditioning Research. 2014;28(6):1649–55. 10.1519/JSC.0000000000000323 24276300

[37] Rhodes J, Mason B, Perrat B, Smith M, Goosey-Tolfrey V. The validity and reliability of a novel indoor player tracking system for use within wheelchair court sports. J Sports Sci. 2014;32(17):1639–47. 10.1080/02640414.2014.910608 24758599

[38] Hoppe MW, Baumgart C, Polglaze T, Freiwald J. Validity and reliability of GPS and LPS for measuring distances covered and sprint mechanical properties in team sports. PLoS One. 2018;13(2):e0192708 10.1371/journal.pone.0192708 29420620 PMC5805339

[39] Beato M, Devereux G, Stiff A. Validity and reliability of global positioning system units (STATSports Viper) for measuring distance and peak speed in sports. The Journal of Strength & Conditioning Research. 2018;32(10):2831–7. 30052603 10.1519/JSC.0000000000002778

[40] Akenhead R, Nassis GP. Training load and player monitoring in high-level football: current practice and perceptions. Int J Sports Physiol Perform. 2016;11(5):587–93. 10.1123/ijspp.2015-0331 26456711

[41] Haugen T, Buchheit M. Sprint running performance monitoring: methodological and practical considerations. Sports Med. 2016;46(5):641–56. 10.1007/s40279-015-0446-0 26660758

[42] Rampinini E, Alberti G, Fiorenza M, Riggio M, Sassi R, Borges TO, et al Accuracy of GPS devices for measuring high-intensity running in field-based team sports. Int J Sports Med. 2015;36(1):49–53. 10.1055/s-0034-1385866 25254901

[43] Stevens TG, de Ruiter CJ, van Niel C, van de Rhee R, Beek PJ, Savelsbergh GJ. Measuring acceleration and deceleration in soccer-specific movements using a local position measurement (LPM) system. Int J Sports Physiol Perform. 2014;9(3):446–56. 10.1123/ijspp.2013-0340 24509777

